# Relationship between employment and mental health outcomes following Cardiac Rehabilitation: an observational analysis from the National Audit of Cardiac Rehabilitation

**DOI:** 10.1016/j.ijcard.2016.06.142

**Published:** 2016-10-01

**Authors:** Alex S. Harrison, Jennifer Sumner, Dean McMillan, Patrick Doherty

**Affiliations:** University of York, Department of Health Sciences, York, UK

**Keywords:** Exercise, Rehabilitation, Coronary disease, Secondary prevention, Employment

## Abstract

**Background:**

Employment status has been shown to impact mental health state and intervention outcomes, yet still to be studied in a Cardiac Rehabilitation (CR) population. This observational study investigated the relationship between employment status and mental health outcomes following Cardiac Rehabilitation (CR).

**Methods:**

All patients with an eligible cardiovascular incident entered into the National Audit of Cardiac Rehabilitation (NACR) 1 January 2013–31st December 2015. Logistic regression comparing the association between employment status and normal mental health categories.

**Results:**

A total of 24,242 CR patients with completed post CR assessments were included and had representative age and gender distribution (mean 65 years, 73.2% male). At baseline the unemployed status had a lower proportion of patients in normal healthy categories than other groups (T-test and chi-squared p = < 0.05). The regression analyses revealed no significant association between retired and employed groups and outcome. There was significant association between unemployed patients and all mental health outcomes except anxiety; all p values < 0.05 and odds ratios between 0.525 and 0.772 showing less likelihood of achieving the normal healthy category.

**Conclusions:**

This is the first UK study, using routinely collected data, to investigate in coronary heart disease patients the impact of employment status on outcomes. The findings were that when weighted for baseline differences, unemployed patients mostly had poorer outcomes. Teams involved in CR delivery should take particular care when interpreting mental health baseline measures when setting CR goals, especially in relation to unemployed patients, and efforts should be made in providing more patient tailored interventions.

## 1 Introduction

Cardiac Rehabilitation (CR) is a highly evidenced based intervention for a variety of cardiac conditions, (1) significantly reducing cardiovascular mortality (RR 0.74, 95% CI 0.64–0.86) and hospital re-admission post CR (RR 0.82, 95% CI 0.70–0.96). [1], [2] The modern United Kingdom (UK) CR population includes patients with conditions such as myocardial infarction, heart failure and angina, along with treatments such as percutaneous coronary intervention, coronary artery bypasses graft and valve surgery. [1] The benefits of CR are derived from modifications to lifestyle risk factors and the management of psycho-social factors associated with well-being. The approach is globally recognised as multi-disciplinary and comprehensive including structured education sessions, exercise based interventions and psychosocial support with agreed core components and minimum standards [3], [4], [5] yet less than 25% of programmes have access to psychosocial services. [6].

Current evidence in a post Percutaneous Coronary Intervention (PCI) population showed a link between employment, specifically unemployment, and lowered quality of life at baseline and 12 months post treatment [7]. This link between employment and health has scarcely been studied in CR, often only in uptake and participation [7], [8], [9], [10], [11] The work by Strens et al. showed employment status at baseline was associated with reduced participation in CR post PCI (OR 0.54 CI 95% 0.44–0.68) or surgical intervention (OR 0.51 CI 95% 0.36–0.73) [8]. A study of patients following myocardial infarction found that unemployment was significantly associated with reduced intention to attend CR (p = 0.007) and increased drop out (p = 0.044) [9]. In a US study of underserved populations, patients were found to be less likely to attend CR if they were unemployed; however, conflict with work has also been identified as a common reason to not complete. [11] Although there is evidence of employment status affecting uptake and completion of CR, there is a dearth of evidence as to whether CR, as an intervention, is as effective in different employment statuses in terms of patient outcome. As such the aim of this study was to ascertain the general patient characteristics by employment status and investigate the association between employment status (employed, unemployed and retired) and patient outcome following CR; specifically mental health and quality of life (QoL).

## 2 Methods

This study was reported according to the Strengthening the Reporting of Observational Studies in Epidemiology (STROBE) guidelines. [13].

### 2.1 Data

The analyses were performed using routinely collected patient level data from the UK NACR database from 1st January 2013 to 31st December 2015. According to the 2015 NACR report a total of 164 CR programmes across the UK enter into the NACR audit [6]. Information on patient's initiating event, treatment, individual risk factors, medication use, characteristics and outcomes of CR users is captured. Data is collected under 251 approvals which are reviewed annually by the Health and Social Care information Centre (HSCIC).

The analysis included all CR programmes in England, with valid patient data at both pre and post CR assessment including deprivation score as measured by the Index of Multiple Deprivation (IMD). Patients who had Myocardial Infarction with or without revascularisation were included to account for type of diagnosis/treatment. All patients with valid diagnosis/treatment entered were included, minimising selection bias.

### 2.2 Cardiac Rehabilitation

CR is conducted according to the British Association for Cardiovascular Prevention and Rehabilitation (BACPR) core components [3]. Typically programmes run for 8–12 weeks, twice weekly with structured education and exercise components.

### 2.3 Employment status

Employment status was categorised as employed, unemployed or retired. Being employed was classified as either full or part time employment, self-employed or as part of a government training scheme. Unemployed was defined as; unemployed, looking after family/home, permanently sick/disabled, temporarily sick or injured, student or other reasons for not working.

Employment status is often defined in a variety of ways, most commonly employed–unemployed comparisons are made sometimes including a third group; such as retired [14]. In the UK CR population the mean age of males is 66 years and females is 70 years, with approximately two thirds of population reported as being retired [6]. As such this study will include three employment groups; employed, unemployed and retired.

### 2.4 Outcome measures

Anxiety and depression symptoms were separately measured on the Hospital Anxiety and Depression Scale (HADS), licensed to NACR, (score range 0–21) with higher scores representing worse symptoms, patients were grouped as healthy normal category (< 8) and unhealthy score (8 +) [15]. Quality of life in relation to feelings and general quality of life were assessed on the Dartmouth COOP (score per item 1–5), responses were dichotomised (healthy normal score 1–3, unhealthy score 4–5) [16].

### 2.5 Statistical Analysis

The analyses were conducted in STATA 13.1. Baseline characteristics were compared across groups using Chi^2^ or T-test as appropriate. Standardised differences were calculated for continuous variables, with > 0.1 classified as meaningful. Unemployed and retired groups were compared to the baseline employed group [16]. Regression models were run comparing the unemployment and retired populations to the reference category employed. Relevant important covariates were included in the analysis. Age (years), gender (male/female) and number of comorbidities have both been shown to influence the outcomes following a variety of different interventions, including CR [17], [18] The duration of CR (length of core rehabilitation) was accounted for in analysis. The type of event/treatment prior to CR is likely to affect the patients' outcomes, to account for this variation patients were coded as medically managed or re-vascularised as shown in the NACR statistics report [6]. The IMD was calculated and ranked, from the most deprived to the least deprived regions, at for all 209 clinical commissioning groups and was included in this analysis [19]. Individual patients were assigned an IMD score according to where their General Practitioner (GP) was located within England. IMD was split into 10 equal sized groups ‘deciles’, with 1 being the most deprived group.

Logistic regressions were used to investigate the association between employment status, as an independent variable, and mental health outcomes as the dependent variable. Significance was set at the p < 0.05 level. Data model checking was performed to ensure that the models were a good fit through assumptions associated with the regressions.

## 3 Results

### 3.1 Study population

**Fig. 1:**
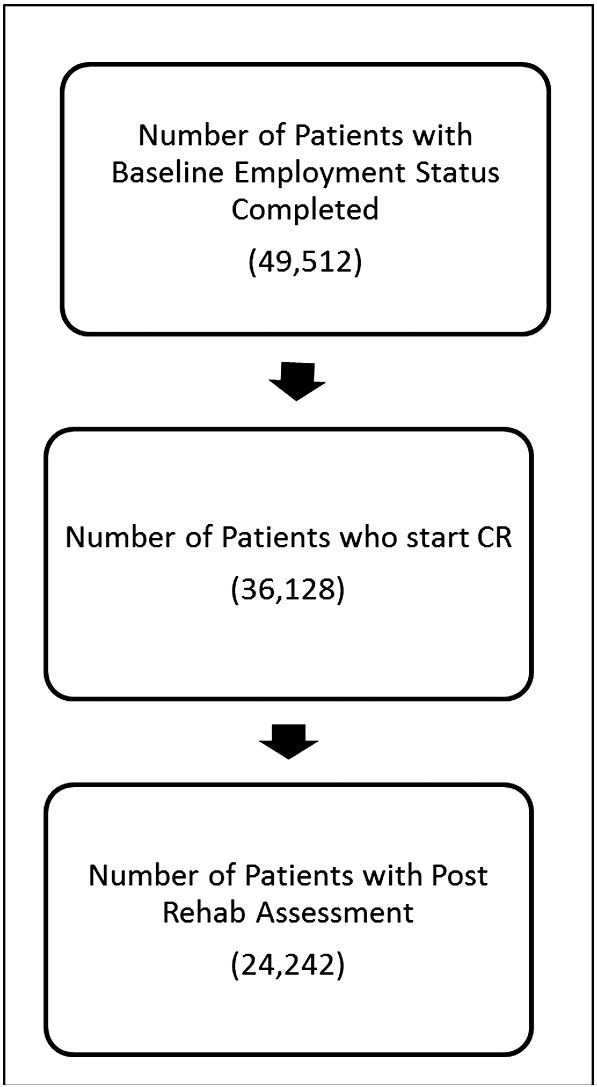
Flow diagram showing patients' numbers from assessment 1 with a valid employment status field, starting core rehabilitation and then a valid assessment 2 post rehabilitation. Of the number with assessment 1 49% go on to have an assessment 2.

The study sample is summarised in Fig. 1 and the population characteristics are summarised in Table 1. A total of 24,242 patients were included in the analyses.

The population is representative of patients accessing CR [6], with an average age of 65 years (SD 11.9) and majority male participants (73.2% male). The average duration of CR for this study falls within the NICE guidelines of 8–12 weeks, with this population averaging 9 weeks. The distribution of the employment statuses is similar to the national level, which has stayed static at 58% retired for the past 6 years [6]. The patients were evenly distributed across the IMD deciles with the highest proportion in the 8th decile.

In terms of baseline scores by employment group, mean HADS were 2 points higher on average in the unemployed group (mean anxiety 7.7, depression 6.4) compared to the other two groups. Overall unemployed patients had the smallest proportion classified as normal on the HADS. The unemployed group also had the smallest proportions of patients reporting normal QoL readings in relation to feelings and general QoL, around 10% lower in comparison. The number of comorbidities was lowest in the employed group and duration of CR was greater, by 4 days, in the unemployed group. Naturally, the age was significantly different in the retired population with a 14 years greater average.

Table 1 also shows the proportion change from baseline to post rehabilitation into the normal group (HADS < 8 and Dartmouth ≤ 3) for the 4 mental health outcomes split by employment status. The results show that all groups had improvements across the four outcome measures, but the largest improvements were observed in the unemployed group.

### 3.2 Outcomes

The results from the regression analyses are presented in Table 2. The results consistently, apart from anxiety, showed that unemployed patients are significantly associated with worse mental health post rehabilitation (all p < 0.05). The depression results showed unemployed patients were 26% less likely to be in the normal category (p < 0.034), and patients were 23–45% less likely to be in the normal category for Dartmouth feelings and QoL (p < 0.001). No significant associations were found between the retired population and mental health outcomes.

## 4 Discussion

The overriding result of this study is that although all employment groups show improvements in all post CR mental health outcomes, when compared to the employed group, unemployed patients were less likely to be in the normal category, post CR, for depression and Dartmouth feelings and QoL. Anxiety was inputted in a model as well, however, no significant association was found despite unemployed patients having a lower percentage in the baseline normal group. Interestingly work by Meyer et al. showed the complexity surrounding anxiety and outcome when they found that some level of anxiety, even as high as ≥ 10 on the HADS score, is associated with a beneficial reduction in cardiovascular events in a subset of cardiac patients undergoing PCI (p = 0.014) [20].

When compared at baseline, unemployed patients' mental health is consistently worse than the employed or retired population. Although the unemployed group make the greatest improvements pre to post CR this is likely due to worse pre CR starting point and some level of the other groups experiencing ceiling effects.

The unemployed patients' at follow-up were significantly (15–26%) less likely to be in the normal category for the HADS Depression and Dartmouth questions; this result was not significantly represented in the anxiety measure.

This seems consistent with the literature, in that unemployment has an association at baseline with poorer mental health [7], [10], [21] The work by Waddell concluded a similar effect of employment status on mental health outcomes, in that unemployed status can be detrimental to mental health [21]. Additionally Brown and Jin's work also showed higher odds of poorer mental health in unemployed patients [12], [22]

To date the literature investigating the effect of employment on CR, has only compared how patients differ at uptake and dropout [8], [9], [10], [11]. This research has extended knowledge on the characteristics of those accessing CR from different employment groups and has identified an association between employment and outcome. In addition to existing research this current study has identified that from initiating event through to completion of CR there is a need for service tailoring to make sure all employment groups benefit from this intervention.

Overall this study enforces the importance of employment status on the CR population. Unemployed patients are less likely to attend CR and when they do attend they are less likely to be in three of the normal mental health outcome groups. This study's results, along with work on attendance and drop out suggest that commissioners may need to look at aligning the recruitment to and the delivery of CR by employment status [8], [9], [10], [11], [12]

### 4.1 Limitations

One limitation of this study is the level of missing data. Although sufficiently powered for the purposes of this analysis, the inclusion of England only patients and ~ 31% missing data at the post rehab assessment may have limited the generalisability of the findings, although the population did appear to be representative of patients accessing CR in the UK. [13].

## 5 Conclusion

This study identified a strong association between employment status and mental health outcomes. The extent of benefit to patients is significantly influenced by employment status in that being unemployed led to reduced benefit in depression and QoL compared to patients who were employed or retired. Existing evidence has already established a link between employment and mental health at baseline; however, this is the first study to show this impact on patient outcomes. As recommended by national associations, CR teams need to assess patients, based on the core components of CR, and consider employment status when tailoring care for individual patients. Future research should consider the staffing profile and types of tailored interventions that would enable unemployment patients to derive the same benefit.

## Conflict of Interest

The authors report no relationships that could be construed as a conflict of interest.

## Funding

This research was carried out by the British Heart Foundation (BHF) Cardiovascular Care and Education Research Group which is supported by a grant from the BHF (R1680901).

## Figures and Tables

**Table 1:** Baseline and change in patient characteristics and outcome measures by employment status.

Baseline characteristics	Employment status groups			
Employed	Unemployed	Retired	Total
Count n (%)	13,820 (27.9)	8253 (16.7)	27,439 (55.4)	49,512**
Male (%)	84.2	73.1	67.7	73.2**
Mean age (SD)	56.1 (9.1)	56.2 (10.3)	72.9 (7.5) ^a^	65.5 (11.9)**
Number of comorbidities (median)	1	2^a^	2 ^a^	2**
Duration of CR days (median)	63	67^a^	63	63**

% in Normal Category				
HADS anxiety mean (%)	69.7	57.9	77.4	72.3**
HADS depression mean (%)	83.8	69.0	83.9	81.7**
Dartmouth feelings (%)	85.0	76.8	88.1	85.4**
Dartmouth quality of life (%)	95.6	91.8	95.6	95.0**


Change from baseline in outcomes	% Change into Normal Category by Employment Status			
Employed	Unemployed	Retired	Total
HADS anxiety (%)	7.1	8.0	4.6	6.1
HADS depression (%)	5.8	8.4	5.3	5.7
Dartmouth feelings (%)	5.9	6.4	4.3	5.3
Dartmouth quality of life (%)	2.6	3.6	2.4	2.6

Standardised differences ^a^ > 0.1 from employed group and Chi Squared * = p < 0.05 and ** = p < 0.001.

**Table 2:** Results from the Multivariate Regression Analysis; association between employment status and mental health outcomes.

	Odds ratio	Sig.	95% CI		Observations
*Effect of being unemployed in comparison to employed*					
HADS anxiety	0.934	0.56	0.743	1.175	23,209
HADS depression	0.734	0.034	0.552	0.977	23,244
Feelings	0.772	< 0.001	0.675	0.884	21,618
Quality of life	0.525	< 0.001	0.406	0.678	21,530

*Effect of being retired in comparison to employed*					
HADS anxiety	0.992	0.98	0.513	1.915	23,244
HADS depression	0.978	0.892	0.711	1.346	23,209
Feelings	0.988	0.872	0.849	1.149	21,618
Quality of life	0.802	0.151	0.593	1.084	21,530
